# 2-(4-Methyl-2-phenyl­piperazin-4-ium-1-yl)pyridine-3-carboxyl­ate dihydrate

**DOI:** 10.1107/S1600536808011288

**Published:** 2008-06-07

**Authors:** Ai-Jun Li, Xiao-Hua Zhang, Wen-Qian Sun, Xue-Qin Zhou, Dong-Zhi Liu

**Affiliations:** a College of Chemical and Pharmaceutical Engineering, Hebei University of Science and Technology, Shijiazhuang 050018, People’s Republic of China; b Department of Chemical and Environmental Engineering, Hebei Chemical and Pharmaceutical College, Shijiazhuang 050026, People’s Republic of China; c School of Chemical Engineering and Technology, Tianjin University, Tianjin 300072, People’s Republic of China

## Abstract

The title compound, C_17_H_19_N_3_O_2_·2H_2_O, is particularly useful in the preparation of mirtaza­pine, which is the active agent in a new class of anti­depressants. It crystallized as a zwitterion with two mol­ecules of water in the asymmetric unit. The crystal structure is dominated by a system of hydrogen bonds involving the positively charged N atom and both water mol­ecules.

## Related literature

For details of the synthesis see: Eiichi *et al.* (2002*a* ▶,**b* ▶)*; Metzger *et al.* (2004 ▶). For related literature, see: Singer *et al.* (2004 ▶).
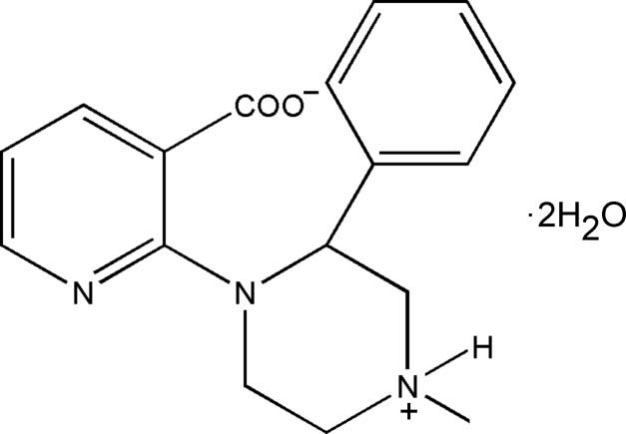


## Experimental

### 

#### Crystal data

C_17_H_19_N_3_O_2_·2H_2_O
*M* *_r_* = 333.38
Monoclinic, 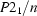

*a* = 12.730 (7) Å
*b* = 8.157 (4) Å
*c* = 16.814 (9) Å
β = 94.031 (10)°
*V* = 1741.6 (16) Å^3^
*Z* = 4
Mo *K*α radiation
μ = 0.09 mm^−1^
*T* = 294 (2) K
0.24 × 0.20 × 0.18 mm

#### Data collection

Bruker SMART CCD area-detector diffractometer
Absorption correction: multi-scan (*SADABS*; Bruker, 2002 ▶) *T*_min_ = 0.978, *T*_max_ = 0.984
9611 measured reflections
3550 independent reflections
2039 reflections with *I* > 2σ(*I*)
*R*_int_ = 0.051

#### Refinement

*R*[*F* ^2^ > 2σ(*F* ^2^)] = 0.043
*wR*(*F* ^2^) = 0.117
*S* = 1.00
3550 reflections
218 parameters
7 restraints
H-atom parameters constrained
Δρ_max_ = 0.18 e Å^−3^
Δρ_min_ = −0.20 e Å^−3^

### 

Data collection: *SMART* (Bruker, 2002 ▶); cell refinement: *SAINT* (Bruker, 2002 ▶); data reduction: *SAINT*; program(s) used to solve structure: *SHELXS97* (Sheldrick, 2008 ▶); program(s) used to refine structure: *SHELXL97* (Sheldrick, 2008 ▶); molecular graphics: *SHELXTL* (Sheldrick, 2008 ▶); software used to prepare material for publication: *SHELXTL*.

## Supplementary Material

Crystal structure: contains datablocks global, I. DOI: 10.1107/S1600536808011288/fl2184sup1.cif

Structure factors: contains datablocks I. DOI: 10.1107/S1600536808011288/fl2184Isup2.hkl

Additional supplementary materials:  crystallographic information; 3D view; checkCIF report

## Figures and Tables

**Table 1:** Hydrogen-bond geometry (Å, °)

*D*—H⋯*A*	*D*—H	H⋯*A*	*D*⋯*A*	*D*—H⋯*A*
N3—H3*A*⋯O1^i^	0.91	1.77	2.681 (2)	178
O3—H3*C*⋯O4^ii^	0.87	1.92	2.781 (3)	179
O4—H4*A*⋯O2^iii^	0.85	1.94	2.761 (2)	162
O3—H3*B*⋯O1	0.87	2.21	3.038 (3)	161
O4—H4*B*⋯N1	0.85	2.23	3.037 (3)	159

Symmetry codes: (i) 
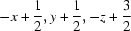
; (ii) 
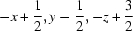
; (iii) 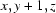.

**Fig. 1.**
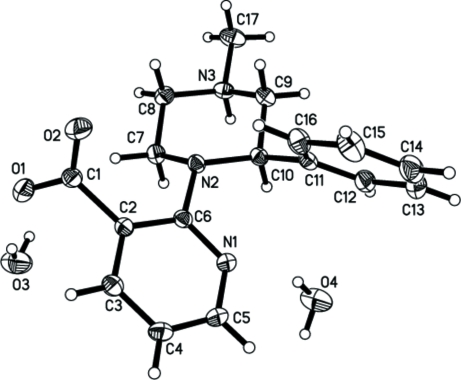
The molecular structure of the title compound, drawn with 30% probability ellipsoids.

**Fig. 2.**
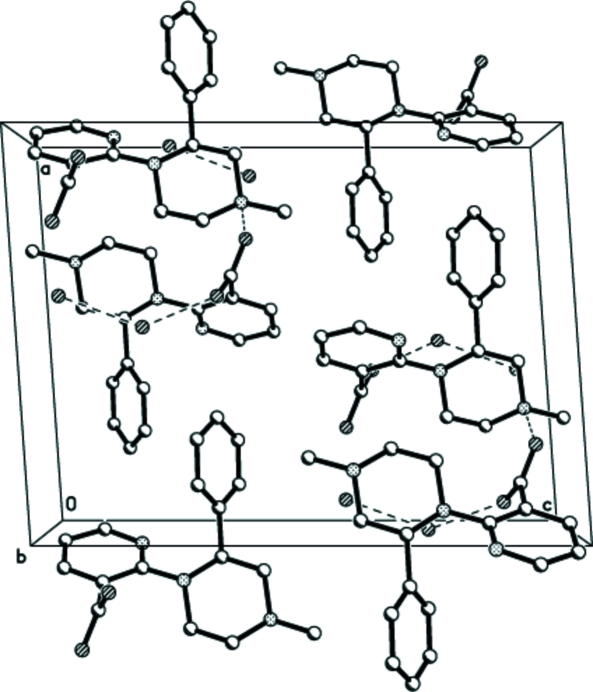
The crystal structure of (I), viewed along a axis

## supplementary crystallographic information

### Comment

The title compound, C_17_H_19_N_3_O_2_.2H_2_O, is particularly useful in the preparation of mirtazapine which is the active agent in a new class of antidepressants. It crystallized as a zwitterion with two molecues of water in the asymmetric unit (Fig 1). The central piperazine ring has a normal chair conformation. In the molecule, the dihedral angle is 81.20 between plane 1(C7 C8 C9 C10) and plane 2(C11 C12 C13 C14 C15 C16), the dihedral angle is 68.40 between plane 2(C11 C12 C13 C14 C15 C16) and plane 3(N1 C2 C3 C4 C5 C6)and the the dihedral angle is 36.90 between plane 1 and plane 3. Packing is dominated by a system of hydrogen bonds involving the positively charged nitrogen and both water molecules (Table 1, Fig. 2)

### Experimental

To 162 g of 1-butanol were added 2-(4-methyl-2-phenylpiperazine-1-yl)pyridine-3-carbonitrile(54 g, 0.2 mol) and 60.93 g of potassium hydroxide. The mixture was heated to 125 - 135 centigrade degree. (Eiichi, *et al.*, 2002*a*; Eiichi, *et al.*, 2002*b*; Metzger, *et al.*, 2004) After 7 h, the reaction mixture was cooled and the butanol removed from the mixture by vacuum distilation after which fresh water and toluene were added and the two phases were separated. The water solution was neutralized with hydrochloric acid to pH=6.5–7. The water was evaporated and toluene was added. The inorganic salt were filtered and toluene solution was evaporated to dryness. Yield:52 g(90%). (Singer *et al.*, 2004) Colourless crystals suitable for X-ray analysis were obtained by slow evaporation of an methanol-toluene solution at room temperature over 30 days.

### Refinement

All H atoms were positioned geometrically and refined as riding, with C—H = 0.93–0.98 Å and N—H=0.949 Å *U*_iso_(H) = 1.2 *U*_eq_(C).

### Crystal data

**Table:**

C_17_H_19_N_3_O_2_·2H_2_O_1_	*F*_000_ = 712
*M**_r_* = 333.38	*D*_x_ = 1.271 Mg m^−^^3^
Monoclinic, *P*2_1_/*n*	Mo *K*α radiation λ = 0.71073 Å
*a* = 12.730 (7) Å	Cell parameters from 1949 reflections
*b* = 8.157 (4) Å	θ = 3.0–22.9º
*c* = 16.814 (9) Å	µ = 0.09 mm^−^^1^
β = 94.031 (10)º	*T* = 294 (2) K
*V* = 1741.6 (16) Å^3^	Block, colorless
*Z* = 4	0.24 × 0.20 × 0.18 mm

### Data collection

**Table:**

Bruker SMART CCD area-detector diffractometer	3550 independent reflections
Radiation source: fine-focus sealed tube	2039 reflections with *I* > 2σ(*I*)
Monochromator: graphite	*R*_int_ = 0.051
*T* = 294(2) K	θ_max_ = 26.5º
φ and ω scans	θ_min_ = 1.9º
Absorption correction: multi-scan(SADABS; Bruker, 2002)	*h* = −14→15
*T*_min_ = 0.978, *T*_max_ = 0.984	*k* = −10→9
9611 measured reflections	*l* = −21→18

### Refinement

**Table:**

Refinement on *F*^2^	Secondary atom site location: difference Fourier map
Least-squares matrix: full	Hydrogen site location: inferred from neighbouring sites
*R*[*F*^2^ > 2σ(*F*^2^)] = 0.044	H-atom parameters constrained
*wR*(*F*^2^) = 0.117	*w* = 1/[σ^2^(*F*_o_^2^) + (0.0459*P*)^2^ + 0.2854*P*] where *P* = (*F*_o_^2^ + 2*F*_c_^2^)/3
*S* = 1.00	(Δ/σ)_max_ = 0.001
3550 reflections	Δρ_max_ = 0.18 e Å^−^^3^
218 parameters	Δρ_min_ = −0.20 e Å^−^^3^
7 restraints	Extinction correction: none
Primary atom site location: structure-invariant direct methods	

### Special details

**Table:**

Geometry. All e.s.d.'s (except the e.s.d. in the dihedral angle between two l.s. planes) are estimated using the full covariance matrix. The cell e.s.d.'s are taken into account individually in the estimation of e.s.d.'s in distances, angles and torsion angles; correlations between e.s.d.'s in cell parameters are only used when they are defined by crystal symmetry. An approximate (isotropic) treatment of cell e.s.d.'s is used for estimating e.s.d.'s involving l.s. planes.
Refinement. Refinement of *F*^2^ against ALL reflections. The weighted *R*-factor *wR* and goodness of fit *S* are based on *F*^2^, conventional *R*-factors *R* are based on *F*, with *F* set to zero for negative *F*^2^. The threshold expression of *F*^2^ > σ(*F*^2^) is used only for calculating *R*-factors(gt) *etc*. and is not relevant to the choice of reflections for refinement. *R*-factors based on *F*^2^ are statistically about twice as large as those based on *F*, and *R*- factors based on ALL data will be even larger.

### Fractional atomic coordinates and isotropic or equivalent isotropic displacement parameters (Å^2^)

**Table:**

	*x*	*y*	*z*	*U*_iso_*/*U*_eq_	
O1	0.23746 (11)	−0.07865 (17)	0.91677 (8)	0.0468 (4)	
O2	0.09369 (12)	−0.19110 (17)	0.85735 (9)	0.0523 (4)	
N1	0.01524 (12)	0.33067 (19)	0.81432 (9)	0.0336 (4)	
N2	0.09094 (11)	0.12896 (18)	0.73567 (8)	0.0276 (4)	
N3	0.18394 (12)	0.11644 (19)	0.58307 (8)	0.0317 (4)	
H3A	0.2110	0.2203	0.5847	0.038*	
C1	0.14487 (17)	−0.0721 (2)	0.88370 (10)	0.0326 (5)	
C2	0.09270 (14)	0.0949 (2)	0.88117 (10)	0.0284 (4)	
C3	0.06312 (16)	0.1573 (2)	0.95305 (11)	0.0377 (5)	
H3	0.0816	0.1015	1.0002	0.045*	
C4	0.00669 (17)	0.3009 (3)	0.95524 (12)	0.0433 (5)	
H4	−0.0156	0.3411	1.0030	0.052*	
C5	−0.01572 (17)	0.3828 (3)	0.88490 (12)	0.0412 (5)	
H5	−0.0543	0.4795	0.8860	0.049*	
C6	0.06542 (14)	0.1873 (2)	0.81196 (10)	0.0276 (4)	
C7	0.20574 (14)	0.1119 (2)	0.72897 (10)	0.0322 (5)	
H7A	0.2362	0.0515	0.7746	0.039*	
H7B	0.2377	0.2198	0.7296	0.039*	
C8	0.23032 (16)	0.0240 (2)	0.65354 (10)	0.0345 (5)	
H8A	0.3060	0.0155	0.6509	0.041*	
H8B	0.2013	−0.0860	0.6535	0.041*	
C9	0.06793 (15)	0.1316 (2)	0.58939 (10)	0.0333 (5)	
H9A	0.0367	0.0231	0.5880	0.040*	
H9B	0.0375	0.1924	0.5439	0.040*	
C10	0.04152 (14)	0.2186 (2)	0.66603 (10)	0.0278 (4)	
H10	0.0702	0.3300	0.6658	0.033*	
C11	−0.07784 (15)	0.2284 (2)	0.66480 (10)	0.0302 (5)	
C12	−0.12829 (16)	0.3603 (3)	0.62727 (11)	0.0396 (5)	
H12	−0.0887	0.4409	0.6042	0.048*	
C13	−0.23716 (18)	0.3743 (3)	0.62352 (13)	0.0502 (6)	
H13	−0.2699	0.4638	0.5981	0.060*	
C14	−0.29645 (18)	0.2561 (3)	0.65735 (13)	0.0530 (6)	
H14	−0.3693	0.2660	0.6556	0.064*	
C15	−0.24739 (17)	0.1225 (3)	0.69391 (13)	0.0530 (6)	
H15	−0.2875	0.0418	0.7164	0.064*	
C16	−0.13856 (16)	0.1075 (3)	0.69733 (11)	0.0419 (5)	
H16	−0.1062	0.0162	0.7215	0.050*	
C17	0.20966 (18)	0.0401 (3)	0.50601 (11)	0.0486 (6)	
H17A	0.1818	−0.0693	0.5028	0.073*	
H17B	0.2847	0.0364	0.5033	0.073*	
H17C	0.1790	0.1040	0.4624	0.073*	
O3	0.40776 (15)	0.1829 (2)	0.92970 (9)	0.0746 (5)	
H3B	0.3615	0.1080	0.9385	0.112*	
H3C	0.4310	0.1736	0.8827	0.112*	
O4	0.01683 (14)	0.6489 (2)	0.72071 (9)	0.0695 (5)	
H4A	0.0391	0.7172	0.7563	0.104*	
H4B	0.0095	0.5500	0.7353	0.104*	

### Atomic displacement parameters (Å^2^)

**Table:**

	*U*^11^	*U*^22^	*U*^33^	*U*^12^	*U*^13^	*U*^23^
O1	0.0447 (10)	0.0401 (9)	0.0542 (9)	0.0108 (7)	−0.0058 (7)	−0.0023 (7)
O2	0.0587 (11)	0.0337 (9)	0.0632 (10)	−0.0008 (8)	−0.0061 (8)	−0.0103 (8)
N1	0.0378 (10)	0.0320 (10)	0.0306 (9)	0.0057 (8)	0.0001 (7)	−0.0031 (7)
N2	0.0271 (9)	0.0327 (9)	0.0230 (8)	0.0018 (7)	0.0017 (6)	−0.0002 (7)
N3	0.0354 (10)	0.0322 (9)	0.0282 (8)	−0.0069 (7)	0.0073 (7)	−0.0036 (7)
C1	0.0419 (13)	0.0324 (12)	0.0242 (10)	0.0035 (10)	0.0065 (9)	0.0006 (9)
C2	0.0286 (11)	0.0301 (11)	0.0264 (10)	0.0013 (8)	0.0006 (8)	−0.0015 (8)
C3	0.0480 (13)	0.0394 (12)	0.0258 (10)	0.0048 (10)	0.0033 (9)	0.0010 (9)
C4	0.0550 (15)	0.0448 (13)	0.0307 (11)	0.0100 (11)	0.0075 (10)	−0.0093 (10)
C5	0.0489 (14)	0.0374 (12)	0.0373 (12)	0.0131 (10)	0.0026 (10)	−0.0088 (10)
C6	0.0261 (10)	0.0290 (11)	0.0275 (10)	−0.0016 (9)	0.0000 (8)	−0.0032 (8)
C7	0.0297 (11)	0.0376 (12)	0.0294 (10)	0.0010 (9)	0.0037 (8)	0.0016 (9)
C8	0.0356 (12)	0.0325 (11)	0.0359 (11)	0.0026 (9)	0.0066 (9)	0.0000 (9)
C9	0.0321 (11)	0.0389 (12)	0.0288 (10)	−0.0060 (9)	0.0027 (8)	−0.0013 (9)
C10	0.0297 (11)	0.0288 (10)	0.0248 (10)	−0.0026 (8)	0.0009 (8)	0.0010 (8)
C11	0.0313 (12)	0.0376 (12)	0.0215 (9)	−0.0016 (9)	−0.0004 (8)	−0.0035 (9)
C12	0.0370 (13)	0.0410 (12)	0.0401 (12)	−0.0013 (10)	−0.0026 (9)	−0.0009 (10)
C13	0.0420 (14)	0.0486 (14)	0.0579 (14)	0.0055 (11)	−0.0114 (11)	−0.0019 (12)
C14	0.0327 (13)	0.0750 (18)	0.0506 (14)	0.0020 (13)	−0.0014 (11)	−0.0101 (13)
C15	0.0366 (14)	0.0773 (18)	0.0455 (13)	−0.0175 (13)	0.0051 (10)	0.0070 (13)
C16	0.0383 (13)	0.0498 (14)	0.0373 (12)	−0.0060 (11)	−0.0002 (9)	0.0063 (10)
C17	0.0517 (14)	0.0610 (15)	0.0350 (12)	−0.0073 (12)	0.0166 (10)	−0.0150 (11)
O3	0.1035 (15)	0.0632 (12)	0.0586 (10)	−0.0064 (11)	0.0167 (9)	−0.0125 (9)
O4	0.0919 (14)	0.0659 (11)	0.0516 (10)	−0.0169 (10)	0.0098 (9)	−0.0118 (9)

### Geometric parameters (Å, °)

**Table:**

O1—C1	1.269 (2)	C9—C10	1.529 (2)
O2—C1	1.234 (2)	C9—H9A	0.9700
N1—C6	1.335 (2)	C9—H9B	0.9700
N1—C5	1.346 (2)	C10—C11	1.520 (3)
N2—C6	1.427 (2)	C10—H10	0.9800
N2—C7	1.480 (2)	C11—C12	1.382 (3)
N2—C10	1.482 (2)	C11—C16	1.389 (3)
N3—C8	1.491 (2)	C12—C13	1.388 (3)
N3—C9	1.493 (2)	C12—H12	0.9300
N3—C17	1.495 (2)	C13—C14	1.373 (3)
N3—H3A	0.9140	C13—H13	0.9300
C1—C2	1.514 (3)	C14—C15	1.379 (3)
C2—C3	1.388 (2)	C14—H14	0.9300
C2—C6	1.409 (2)	C15—C16	1.388 (3)
C3—C4	1.376 (3)	C15—H15	0.9300
C3—H3	0.9300	C16—H16	0.9300
C4—C5	1.371 (3)	C17—H17A	0.9600
C4—H4	0.9300	C17—H17B	0.9600
C5—H5	0.9300	C17—H17C	0.9600
C7—C8	1.509 (2)	O3—H3B	0.8682
C7—H7A	0.9700	O3—H3C	0.8664
C7—H7B	0.9700	O4—H4A	0.8512
C8—H8A	0.9700	O4—H4B	0.8504
C8—H8B	0.9700		
			
C6—N1—C5	118.28 (16)	H8A—C8—H8B	108.2
C6—N2—C7	112.85 (13)	N3—C9—C10	112.07 (14)
C6—N2—C10	115.78 (14)	N3—C9—H9A	109.2
C7—N2—C10	110.66 (13)	C10—C9—H9A	109.2
C8—N3—C9	108.89 (14)	N3—C9—H9B	109.2
C8—N3—C17	112.30 (16)	C10—C9—H9B	109.2
C9—N3—C17	111.98 (14)	H9A—C9—H9B	107.9
C8—N3—H3A	108.5	N2—C10—C11	113.90 (14)
C9—N3—H3A	107.1	N2—C10—C9	109.32 (15)
C17—N3—H3A	107.9	C11—C10—C9	106.98 (14)
O2—C1—O1	125.09 (19)	N2—C10—H10	108.8
O2—C1—C2	118.56 (18)	C11—C10—H10	108.8
O1—C1—C2	116.26 (18)	C9—C10—H10	108.8
C3—C2—C6	117.18 (17)	C12—C11—C16	118.62 (19)
C3—C2—C1	116.78 (16)	C12—C11—C10	118.64 (17)
C6—C2—C1	125.90 (16)	C16—C11—C10	122.70 (18)
C4—C3—C2	120.64 (18)	C11—C12—C13	121.0 (2)
C4—C3—H3	119.7	C11—C12—H12	119.5
C2—C3—H3	119.7	C13—C12—H12	119.5
C5—C4—C3	118.00 (18)	C14—C13—C12	120.0 (2)
C5—C4—H4	121.0	C14—C13—H13	120.0
C3—C4—H4	121.0	C12—C13—H13	120.0
N1—C5—C4	123.40 (19)	C13—C14—C15	119.7 (2)
N1—C5—H5	118.3	C13—C14—H14	120.2
C4—C5—H5	118.3	C15—C14—H14	120.2
N1—C6—C2	122.34 (16)	C14—C15—C16	120.5 (2)
N1—C6—N2	117.30 (15)	C14—C15—H15	119.8
C2—C6—N2	120.36 (16)	C16—C15—H15	119.8
N2—C7—C8	111.90 (14)	C15—C16—C11	120.2 (2)
N2—C7—H7A	109.2	C15—C16—H16	119.9
C8—C7—H7A	109.2	C11—C16—H16	119.9
N2—C7—H7B	109.2	N3—C17—H17A	109.5
C8—C7—H7B	109.2	N3—C17—H17B	109.5
H7A—C7—H7B	107.9	H17A—C17—H17B	109.5
N3—C8—C7	109.50 (16)	N3—C17—H17C	109.5
N3—C8—H8A	109.8	H17A—C17—H17C	109.5
C7—C8—H8A	109.8	H17B—C17—H17C	109.5
N3—C8—H8B	109.8	H3B—O3—H3C	111.9
C7—C8—H8B	109.8	H4A—O4—H4B	117.0
			
O2—C1—C2—C3	106.1 (2)	C17—N3—C8—C7	177.34 (15)
O1—C1—C2—C3	−70.7 (2)	N2—C7—C8—N3	59.1 (2)
O2—C1—C2—C6	−69.5 (3)	C8—N3—C9—C10	58.5 (2)
O1—C1—C2—C6	113.8 (2)	C17—N3—C9—C10	−176.74 (16)
C6—C2—C3—C4	2.0 (3)	C6—N2—C10—C11	−55.0 (2)
C1—C2—C3—C4	−174.01 (19)	C7—N2—C10—C11	175.00 (15)
C2—C3—C4—C5	−2.4 (3)	C6—N2—C10—C9	−174.59 (14)
C6—N1—C5—C4	3.7 (3)	C7—N2—C10—C9	55.40 (18)
C3—C4—C5—N1	−0.5 (3)	N3—C9—C10—N2	−57.03 (19)
C5—N1—C6—C2	−4.1 (3)	N3—C9—C10—C11	179.19 (15)
C5—N1—C6—N2	175.71 (16)	N2—C10—C11—C12	151.89 (16)
C3—C2—C6—N1	1.4 (3)	C9—C10—C11—C12	−87.2 (2)
C1—C2—C6—N1	176.95 (18)	N2—C10—C11—C16	−30.5 (2)
C3—C2—C6—N2	−178.48 (16)	C9—C10—C11—C16	90.4 (2)
C1—C2—C6—N2	−2.9 (3)	C16—C11—C12—C13	1.4 (3)
C7—N2—C6—N1	117.95 (18)	C10—C11—C12—C13	179.18 (17)
C10—N2—C6—N1	−11.0 (2)	C11—C12—C13—C14	0.0 (3)
C7—N2—C6—C2	−62.2 (2)	C12—C13—C14—C15	−1.0 (3)
C10—N2—C6—C2	168.87 (15)	C13—C14—C15—C16	0.6 (3)
C6—N2—C7—C8	170.43 (15)	C14—C15—C16—C11	0.8 (3)
C10—N2—C7—C8	−58.0 (2)	C12—C11—C16—C15	−1.8 (3)
C9—N3—C8—C7	−58.05 (19)	C10—C11—C16—C15	−179.48 (18)

### Hydrogen-bond geometry (Å, °)

**Table:**

*D*—H···*A*	*D*—H	H···*A*	*D*···*A*	*D*—H···*A*
N3—H3A···O1^i^	0.91	1.77	2.681 (2)	178
O3—H3C···O4^ii^	0.87	1.92	2.781 (3)	179
O4—H4A···O2^iii^	0.85	1.94	2.761 (2)	162
O3—H3B···O1	0.87	2.21	3.038 (3)	161
O4—H4B···N1	0.85	2.23	3.037 (3)	159

Symmetry codes: (i) −*x*+1/2, *y*+1/2, −*z*+3/2; (ii) −*x*+1/2, *y*−1/2, −*z*+3/2; (iii) *x*, *y*+1, *z*.
